# Supplementary figures and images for: Auditory cortical activity elicited by infrared laser irradiation from the outer ear in Mongolian gerbils
Source: PLoS One. 2020 Oct 15;15(10):e0240227. doi: 10.1371/journal.pone.0240227 (PMC7561108; doi:10.1371/journal.pone.0240227)

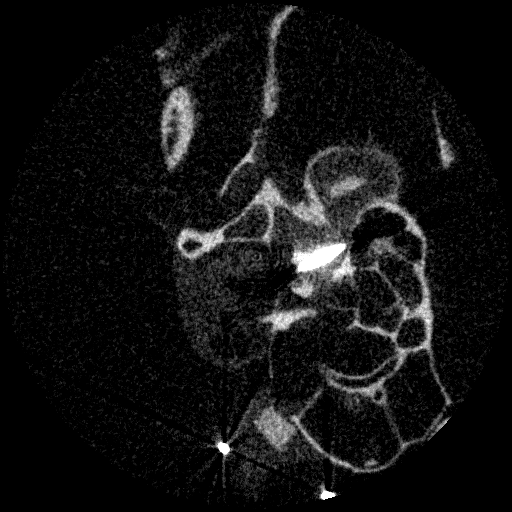

Supplement: S1 File — (ZIP) [file pone.0240227.s001.zip › Submission2020-06-04/Data set for Figure 1A/6.1 mm from the sagittal suture.tif]

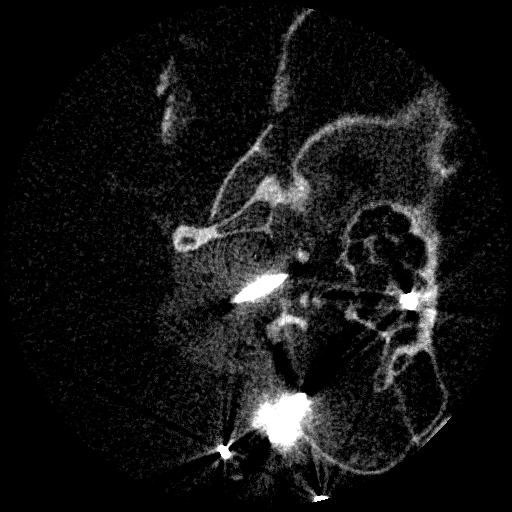

Supplement: S1 File — (ZIP) [file pone.0240227.s001.zip › Submission2020-06-04/Data set for Figure 1A/6.6 mm from the sagittal suture.tif]

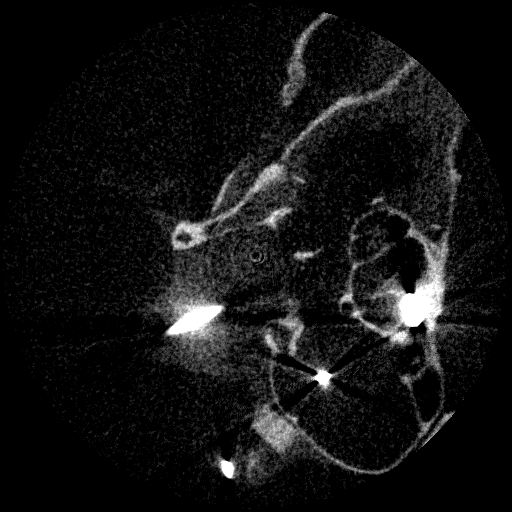

Supplement: S1 File — (ZIP) [file pone.0240227.s001.zip › Submission2020-06-04/Data set for Figure 1A/7.2 mm from the sagittal suture.tif]
