# Supplementary material for: Auditory cortical activity elicited by infrared laser irradiation from the outer ear in Mongolian gerbils
Source: PLoS One. 2020 Oct 15;15(10):e0240227. doi: 10.1371/journal.pone.0240227 (PMC7561108; doi:10.1371/journal.pone.0240227)
Supplement: Attachment — Submitted filename: revision_PONE-D-20-17518.docx [file pone.0240227.s002.docx]

I have read the manuscript entitled “Auditory cortical activity elicited by infrared irradiation from the outer ear in Mongolian gerbils” by Tamai et al. The paper explores the effects of cochlear laser stimulation on the auditory cortex and overall, it is well-written and easy to read. In summary, the authors used brain imaging of activity-dependent changes in mitochondrial flavoprotein fluorescence signal to show that cochlear laser stimulation activates the primary auditory cortex. In addition, they showed that cortical activity evoked by laser stimulation is similar to that evoked by acoustic stimulation. I believe this paper to be highly relevant for PLoS One. The paper is, overall, written concisely and it needs only a few corrections or improvements, which are listed below.

Introduction:

Page 4. Line 60. It would be helpful to the reader if the author summarized the results by Moreno et al. 2011 and Richter et al. 2011 in the inferior colliculus.

Results:

Page 6. Line 83. The data presented in Figure 1is not well described in the text. Please, provide a better description of Fig. 1. For example, it is hard for a general reader to see the cochlear microphonic observed only with the auditory stimuli. The authors should provide a statistical comparison between the cochlear responses to laser and auditory stimuli.

Page 6. Line 86. Define region of interest (ROI) at the first use.

Discussion:

Page 29. Line 141-147. Move to Results section.

The authors should briefly discuss the effects of anesthesia on the temporal characteristics of the cortical activity.
